# Digital Interventions for Self-Management of Type 2 Diabetes Mellitus: Systematic Literature Review and Meta-Analysis

**DOI:** 10.2196/55757

**Published:** 2024-07-22

**Authors:** David Kerr, David Ahn, Kayo Waki, Jing Wang, Boris Breznen, David C Klonoff

**Affiliations:** 1 Center for Health Systems Research Sutter Health Walnut Creek, CA United States; 2 Mary & Dick Allen Diabetes Center at Hoag Newport Beach, CA United States; 3 The University of Tokyo Tokyo Japan; 4 Florida State University College of Nursing Tallahassee, FL United States; 5 Evidinno Outcomes Research Inc Vancouver, BC Canada; 6 Diabetes Research Institute Mills-Peninsula Medical Center San Mateo, CA United States

**Keywords:** coaching, digital health, eHealth, meta-analysis, patient empowerment, patient engagement, self-care, systematic review, telemedicine, type 2 diabetes, digital interventions, self-management, systematic literature review, effectiveness, efficacy, safety, meta-regression

## Abstract

**Background:**

The proliferation of digital technology has the potential to transform diabetes management. One of the critical aspects of modern diabetes management remains the achievement of glycemic targets to avoid acute and long-term complications.

**Objective:**

This study aims to describe the landscape of evidence pertaining to the relative effectiveness or efficacy and safety of various digital interventions for the self-management of type 2 diabetes mellitus (T2DM), with a primary focus on reducing glycated hemoglobin A_1c_ (HbA_1c_) levels.

**Methods:**

A systematic literature review (SLR) was conducted by searching Embase, MEDLINE, and CENTRAL on April 5, 2022. Study selection, data extraction, and quality assessment were performed by 2 independent reviewers. Eligibility criteria for the SLR included randomized controlled trials (RCTs) and comparative observational studies evaluating interventions containing both human (eg, coaching) and digital components (eg, glucose meter) in adult patients with T2DM. The primary meta-analysis was restricted to studies that reported laboratory-measured HbA_1c_. In secondary analyses, meta-regression was performed with the intensity of coaching in the digital intervention as a categorical covariate.

**Results:**

In total, 28 studies were included in this analysis. Most studies (23/28, 82%) used the reduction of HbA_1c_ levels as the primary end point, either directly or as a part of a multicomponent outcome. In total, 21 studies reported statistically significant results with this primary end point. When stratified into 3 intervention categories by the intensity of the intervention supporting the digital health technology (analyzing all 28 studies), the success rate appeared to be proportional to the coaching intensity (ie, higher-intensity studies reported higher success rates). When the analysis was restricted to RCTs using the comparative improvement of HbA_1c_ levels, the effectiveness of the interventions was less clear. Only half (12/23, 52%) of the included RCTs reported statistically significant results. The meta-analyses were broadly aligned with the results of the SLR. The primary analysis estimated a greater reduction in HbA_1c_ associated with digital interventions compared with usual care (–0.31%, 95% CI –0.45% to –0.16%; *P*<.001). Meta-regression estimated reductions of –0.45% (95% CI –0.81% to –0.09%; *P*=.02), –0.29% (95% CI –0.48% to –0.11%; *P*=.003), and –0.28% (95% CI –0.65% to 0.09%; *P*=.20) associated with high-, medium-, and low-intensity interventions, respectively.

**Conclusions:**

These findings suggest that reducing HbA_1c_ levels in individuals with T2DM with the help of digital interventions is feasible, effective, and acceptable. One common feature of effective digital health interventions was the availability of timely and responsive personalized coaching by a dedicated health care professional.

## Introduction

Digital health and telemedicine acceptance is rapidly growing, accelerated by the COVID-19 pandemic restrictions. Although it is difficult to estimate the acceptance of digital health in people with type 2 diabetes mellitus (T2DM) for methodological reasons [1], patients have access to a growing number of digital health technologies to support self-management of their condition. A recent study in Italy found that more than 70% of participants use continuous glucose systems [2]. The concept of self-management as an important part of long-term management of chronic diseases is gaining acceptance, and it is now considered essential for achieving long-term improvement in health outcomes and quality of life [3]. Compared with traditional approaches focused on managing a specific disease condition, the new paradigm is based on a patient-provider partnership involving collaborative care and education in chronic disease self-management [3]. Transition to this new paradigm has been increasingly important for patients with T2DM. A recent survey showed that the standard of care in T2DM, although generally acceptable, cannot meet the variety of patients’ needs in terms of accessibility and timeliness of psychological, emotional, and behavioral support [4]. This unmet need can be alleviated by a wider use of digital technologies designed to help patients with their lifestyle and health-related decisions by making accessible critical data and on-demand consultations [5]. The technologies for managing T2DM include medical devices such as glucose meters, insulin pumps, continuous glucose monitors, and connected insulin pens; digital interventions including mobile apps, SMS text messaging, electronic communications, and videoconference platforms; and wearable technologies for monitoring health, such as activity trackers, sleep trackers, and smartwatches [6,7]. Digital health technologies can also support digital health care services, outside of a clinic or office, by using remotely collected data and communication capabilities of mobile phone devices and the internet [8]. The specific form of remote care can vary significantly: from occasional automated text messages to real-time teleconferencing with a dedicated health care professional (HCP). The intensity of remote care is therefore one of the factors that may impact the success of the interventions.

One of the critical goals of modern diabetes management remains the achievement of acceptable levels of glycemia to avoid the acute and long-term complications associated with T2DM [9]. Unfortunately, many individuals do not achieve their preferred glycemic targets or experience unwanted glycemic variability [10]. It has been suggested that digital technology has the potential to support people living with T2DM in their efforts toward achieving their glycemic goals [11,12]. A core need within diabetes self-management is to provide actionable information based on measured glucose levels [13]. This can be accomplished with timely information and possibly additional support from HCPs [8]. The advantages of digital technologies in managing glucose levels from patient’s perspective were recently summarized in 3 essential concepts: competence, autonomy, and connectivity [14]. Competence refers to the understanding of the blood glucose levels with the help of supporting apps, autonomy means that the digital interventions allow for independent and timely decisions, and connectivity means that an HCP is always available through text messages or email. Digital interventions provide for all 3 components mentioned here, and thus, they are empowering the patients in their effort to cope with the disease. However, research suggests that people with T2DM may need more than knowledge about healthy eating, exercise, and self-monitoring of blood glucose [15]. They also need assistance in building insights into their daily health-related behaviors and routines [16,17].

The aim of this systematic literature review (SLR) and meta-analysis was to analyze digital health interventions for diabetes stratified by the levels of intensity of the intervention to determine whether (1) digital health interventions for diabetes are associated with improved outcomes and (2) whether the intensity of the intervention affects the degree of improvement. Additional outcomes of interest included user engagement measured by adherence or persistence, retention, and study withdrawal rates.

## Methods

### Eligibility Criteria

An SLR was undertaken following the standard methodologies for conducting and reporting systematic reviews as recommended by the Cochrane Handbook for Systematic Reviews of Interventions and the PRISMA (Preferred Reporting Items for Systematic Reviews and Meta-Analyses) guidelines [18,19]. Study eligibility criteria were defined using the PICO (Population, Intervention, Comparator, and Outcomes) framework (Multimedia Appendix 1). Briefly, eligibility criteria for the SLR included randomized controlled trials (RCTs) and comparative observational studies evaluating interventions containing both human (eg, coaching) and digital components (eg, glucose meter) in adults (>18 years) with T2DM.

Two independent reviewers were responsible for reviewing all records, inclusive of conference proceedings and gray literature sources, at the title and abstract stage according to the predefined selection criteria. The eligible studies identified during the title and abstract screening proceeded to the full-text screening stage, where they were assessed for eligibility by the same reviewers. During each of the previous 2 screening stages, reviewers reconciled differences between their decisions, and in scenarios of unresolved discrepancies, a third reviewer intervened to reach a consensus.

Studies that matched the PICO criteria following the full-text screening were included for data extraction. A standardized data extraction table was generated to define the study characteristics, including participant characteristics, intervention characteristics, and outcomes from eligible studies. Two independent reviewers extracted all relevant data from the final list of included studies. The reviewers reconciled discrepancies between their data extraction, and in scenarios of unresolved discrepancies, a third reviewer intervened to reach a consensus.

### Information Sources

Relevant studies were identified by searching the following databases on April 5, 2022: Embase (Multimedia Appendix 2), MEDLINE (Multimedia Appendix 3), and CENTRAL (Multimedia Appendix 4). Abstracts from relevant conferences held between 2018 and 2022 were also searched via Embase or their respective websites. Additionally, selected company websites (Dario, Lark, Livongo, Omada, Onduo, OneDrop, Vida, Virta, and Welldoc) and the US clinical trials registry (ClinicalTrials.gov) were searched.

### Risk of Bias

For quality control, 2 independent reviewers assessed the quality of the included studies using the Cochrane risk of bias tool for RCTs and the Risk of Bias in Nonrandomized Studies–Interventions tools. A third investigator intervened to reach a consensus if there were any unresolved conflicts. Results were summarized in a narrative form.

### Synthesis of Results (Qualitative)

Following the study selection, the results were summarized by grouping the interventions into 3 broad categories as described below. The 2 main components present in all interventions were the technological (devices and software) and the human (coaching). The coaching sessions varied markedly in terms of their frequency (how often the HCPs communicated with the individual with T2DM), duration (both duration of the individual sessions and overall duration of coaching), mode of communication (in person, videoconferencing, phone calls, and SMS text messaging), and the content (personalized vs generic). Categories were created by considering the features and intensity of the coaching component, and for categorization, the intervention had to meet most of the following criteria:

- High intensity: Participant data are automatically uploaded to the cloud at regular intervals. The coaching includes personalized motivational and goal-setting components based on the most recent data and is delivered by dedicated HCP staff. Communication happens regularly, either in person or remotely, at least once per week. Education includes specific modules explaining disease, behavioral strategies, and psychological coping.
- Medium intensity: Participant data are manually uploaded to the clinic database. Coaching includes personalized advice based on individual data but does not include behavioral advice in terms of motivational and goal-setting components. The communication is ad hoc and initiated by the HCPs. Education includes general information about the disease and technical information about the use of the devices.
- Low intensity: Participant data sharing is limited (eg, patients brought the glucose meters to the center, or a nurse visited patients), and the feedback is generic, often using preexisting templates. The communication is asynchronous or delayed (eg, email or follow-up phone call). There is limited or no education.

In addition to separating the studies into the 3 categories of intervention intensity, 3 additional features of the coaching were identified as potentially relevant to the success of an intervention:

- Communication mode—synchronous versus asynchronous: Synchronous mode meant that participants were in direct contact in real time with the HCP or the coach (eg, a telephone call or a teleconference) [20]. Asynchronous communications usually involved web-based portals, emails, or SMS text messages [21].
- Frequency of communication: this varied considerably across the studies, and therefore, the final binary classification was chosen to be unlimited communications [22], or restricted or scheduled communications [23].
- Qualification of the coaches: diabetes specialists (eg, certified diabetes educators [24] or diabetes nurse educators [25]) versus general HCPs (such as general practitioners [26] or study nurses [27]).

### Synthesis of Results (Meta-Analysis)

Nine random effects meta-analyses were conducted on the mean difference (MD) in the change in glycated hemoglobin A_1c_ (HbA_1c_). The primary analysis comprised all the included RCTs with laboratory-measured HbA_1c_ levels and was performed both with and without intervention intensities as categorical covariates via a meta-regression. Subgroup analyses were conducted for high-intensity (number of studies, *k*=4), medium-intensity (*k*=12), high- and medium-intensity (*k*=16), and low-intensity (*k*=4) interventions. Sensitivity analyses were conducted (1) including studies with nonlaboratory measured HbA_1c_, (2) excluding studies with continuous glucose monitoring (CGM), and (3) excluding studies identified as posing a high risk of bias using the Cochrane risk of bias tool.

All meta-analyses were conducted in R (version 4.1.1, The R Foundation for Statistical Computing) using the *metafor* (version 3.0-2) package. The restricted maximum likelihood estimator was used to measure the between-study variance (τ^2^) as heterogeneity due to variation in intervention design, follow-up time, and clinical population across the evidence base was anticipated. We also report the estimated heterogeneity using *Q* and *I*^2^. If more than 1 usual care arm was present within a single study, then they were pooled into 1 sample size weighted mean prior to the meta-analysis. In cases where a study reported multiple timepoints, the final time point was chosen for analysis.

## Results

### SLR Results

#### Study Selection and Study Characteristics

**Figure 1:**
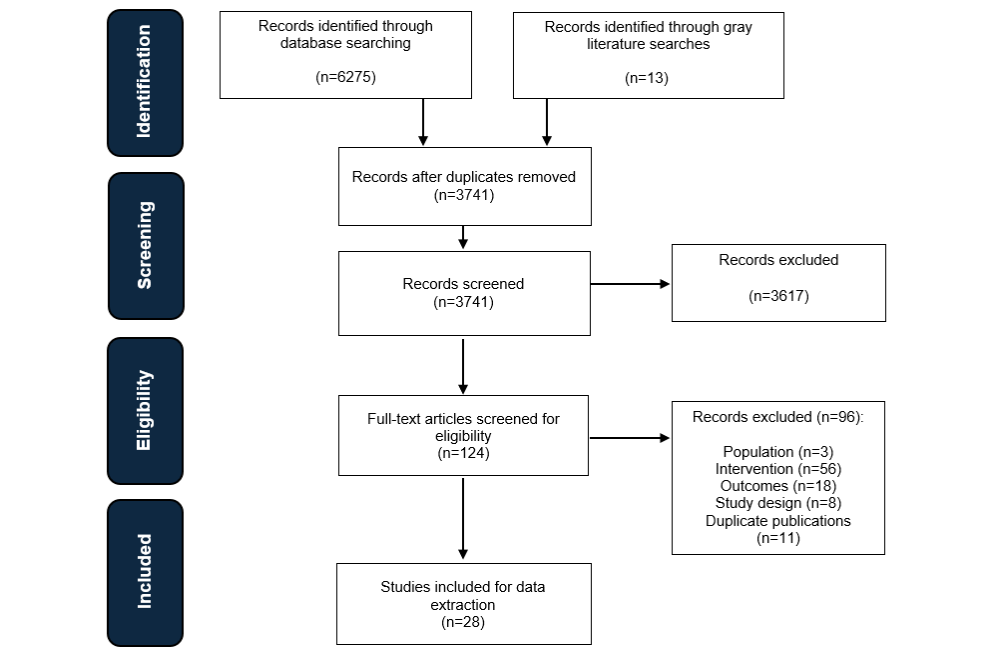
PRISMA (Preferred Reporting Items for Systematic Reviews and Meta-Analyses) diagram.

In total, 6288 papers were identified from the SLR, including 6275 papers via Embase, MEDLINE, and CENTRAL and 13 additional papers through conference proceedings and company websites. After title and abstract screening and full-text screening, a total of 28 studies [20-47] were included in the SLR (Figure 1).

Of the 28 included studies, 23 (82%) were RCTs [20-30,32-34,36-43,45]; 2 (7%) were nonrandomized comparative [31,46]; and the remaining 3 (11%) were cross-sectional [44], prospective cohort [47], and retrospective cohort [35] studies. There were 9 countries where the studies were conducted: the United States (n=12); South Korea (n=6); the United Kingdom (n=3); China (n=2); and 1 each in Belgium, Canada, France, India, and Malaysia. The studies were published between 2003 and 2021. The study population ranged from a minimum of 17 [42] to 772 [35], with an average population size of 202 and a median of 143 participants.

Follow-up durations ranged from 1 month [26] to 24 months [31], with an average follow-up duration of 7.9 months and a median of 6 months (1 abstract did not include information on the follow-up duration [44]).

#### Participant Characteristics

All studies enrolled populations with T2DM, but some studies targeted subpopulations of individuals with T2DM that met specific criteria. A total of 4 studies enrolled only participants taking glucose-lowering prescription medications [24,37,39,42]. Individuals with suboptimally controlled diabetes were investigated by 5 studies [27,29,35,41,44]. The definition of “suboptimally controlled” varied. In some cases, the criterion was set by quantitative thresholds: 2 consecutive HbA_1c_ recordings greater than 8% in the previous 12 months [29,41] or HbA_1c_≥7.5% to ≤13% [27]. However, in 2 studies, the definition was only descriptive with no quantitative data [35,44]. Four studies targeted populations with low income or low socioeconomic status [20,22,32,38]. Out of the 4 studies, 2 (50%) studies enrolled only individuals who were overweight (BMI≥25) [31] or obese (BMI≥41) [46]. The 2 studies selected participants from a pool of insured patients [30,45]. One study enrolled only women with T2DM [36], and another study enrolled self-described physically inactive individuals [34]. The mean age of the participants ranged from 47.3 [40] to 64 years [24], with an overall mean age of 55.7 years, a median age of 54.3 years, and 51% (range 29%-100%) being female. Ethnicities and races in the studies included Black, Chinese, Korean, and White. A total of 11 studies reported the baseline average disease duration from 2.6 [42] to 14 years [23], with an overall mean of 7.9 years and a median of 8 years.

All included studies reported the average (or median) level of HbA_1c_ at baseline, and all studies tested disparities between intervention groups. None of them reported significant differences in baseline HbA_1c_ levels. The overall average baseline HbA_1c_ levels varied from 6.8% [40] to 10.9% [39], with an overall mean of 8.6% and median of 8.5%. The distribution of the mean baseline HbA_1c_ across the studies was as follows (Multimedia Appendix 5): ≤7% (2 studies), >7% and ≤8% (7 studies), >8% and ≤9% (10 studies), and >9% (9 studies).

#### Intervention Characteristics

The technology component of the interventions included technology for measuring glucose: either CGM (5 studies) [26,35,36,43,47], self-monitoring of blood glucose (21 studies) [20-24,27-33,37-42,44-46], or both [25]. An exception was a study measuring blood glucose in a clinical setting [34]. In addition to glucose-monitoring devices, several studies used connected scales [31,46] for weight monitoring or accelerometers to monitor physical activity [21,25,30,34,36,41]. The glucose data were usually uploaded to a central server and then used by HCPs to adjust the treatment regimen and to coach or advise the participants on appropriate actions. The information about the measured glucose levels was generally available to participants either directly through the device display or sometimes through a visualization software application or a dedicated website. Additional details on the digital interventions, the usual care groups, and coaching components of the included studies are summarized in Multimedia Appendix 6 [20-47].

#### Outcome Characteristics

Most studies reported improvement in glycemic control in patients with T2DM using HbA_1c_ levels as the primary end point either directly or as a part of a multicomponent outcome. A few studies used feasibility, acceptability, and self-efficacy of the intervention as their end point [36,43,47], and 1 study used a physical activity end point [34]. The breakdown of study end points and the respective number of significant results is summarized in Table 1.

The classification of the studies into the three categories of interventions as outlined in the *Methods* section yielded the following stratification: (1) a total of 7 studies in the high-intensity category [20,22,26,35,41,45,46] (out of which 5 were RCTs) [20,22,26,41,45], (2) a total of 16 studies in the medium-intensity category [21,23,25,27,29-33,37-40,42-44] (out of which 14 were RCTs) [21,23,25,27,29,30,32,33,37-40,42,43], and (3) a total of 5 studies in the low-intensity category [24,28,34,36,47] (out of which 4 were RCTs) [24,28,34,36].

Table 2 shows reported outcomes across the 3 categories as measured by the number of significant primary end points (across all 28 studies and across the 23 RCTs, respectively).

Table 3 summarizes the reported successes in the comparative reduction of HbA_1c_ across the 3 intervention categories within the included RCTs. The data show the number of studies reporting a statistically significant difference in HbA_1c_ reduction between the intervention arm and the comparator arm.

Table 4 shows the summary of the successes in the comparative reduction of HbA_1c_ separated into the categories outlined earlier. Only results from RCTs are included.

Participant engagement and satisfaction were investigated in 3 studies based on their involvement in the counseling and educational sessions [36], reported measurement of the burden [47], or using validated questionnaires targeting self-care and self-efficacy [43]. The tools by which the studies measured some aspects of participant satisfaction consisted of standardized questionnaires and exit interviews. In the studies investigating user engagement, significant differences between the intervention and usual care groups in terms of changes in self-care behaviors were observed. Overall, digital interventions were well received with high completion rates (most of the studies had dropout rates below 20%) and acceptable additional burden to the patients. In the studies investigating satisfaction using the Diabetes Treatment Satisfaction Questionnaire (DTSQ), 2 (67%) out of 3 studies reported significant improvement in DTSQ scores in the digital intervention groups, compared with usual care. One study reported DTSQ improvement from 31.9 (SD 10.1) to 42.0 (SD 3.8) points (*P*<.001) in the intervention group, and from 34.3 (SD 8.5) to 36.4 (SD 8.9) points in the control group (*P*=.10). This difference between the 2 groups was significant (*P*=.01) [39]. In the second study, DTSQs showed a significant rise only in the intervention group, resulting in a 2.21-point increase in the intervention group compared with the control group at 3 months (*P*=.01) [33].

Another aspect of user engagement can be inferred from dropout rates. A total of 25 studies reported dropout rates (defined as the number of participants enrolling in the program but not finishing for any reason). Most studies reported ≤20% dropout, with rates of >30% reported in only 3 studies [20,27,45].

A total of 11 (39%) of the 28 included studies reported on adverse events. Seven of these studies reported no intervention-related adverse events [22,25,27,37,38,45,46]. Four studies reported the occurrence of adverse events without commenting on their relationship to digital interventions [30,31,33,42]. The most common adverse event reported in these 4 studies was hypoglycemia, followed by cardiovascular events, cancer, and metabolic disruptions. Adverse events were reported in both the intervention groups and the usual care groups. The authors of these studies were agnostic about the causal relationship between digital interventions and adverse events.

**Table 1:** Study end points and reported results.

Primary end point	Studies (N=28), n (%)	Studies with significant results^a^ (n=21), n (%)
Change in HbA_1c_^b^	19 (68)	14 (67)
Multicomponent outcomes including HbA_1c_^c^	4 (14)	4 (19)
HOMA2-IR^d^	1 (4)	1 (5)
Feasibility, acceptance, and self-efficacy	3 (11)	2 (10)
Physical activity	1 (4)	0 (0)
Total	28 (100)	21 (100)

^a^Number of studies achieving statistically significant results in primary end point.

^b^HbA_1c_: glycated hemoglobin A_1c_.

^c^Multiple primary end points: HbA_1c_, glycemic control (HOMA2-IR, glycemic variability, fasting blood glucose, and postprandial 2-hour blood glucose), medication use, BMI, weight control, and retention rate.

^d^HOMA2-IR: Homeostatic Model Assessment of Insulin Resistance.

**Table 2:** Success rate in achieving its predetermined primary end point across the 3 intervention categories.

Intervention category	Studies (N=28), n (%)	Significant end points^a^, n/N (%)	RCTs^b^ (n=23), n (%)	Significant end points^c^, n/N (%)
High intensity	7 (25)	6/7 (86)	5 (22)	4/5 (80)
Medium intensity	16 (57)	12/16 (75)	14 (61)	10/14 (71)
Low intensity	5 (18)	3/5 (60)	4 (17)	3/4 (75)

^a^Number of studies achieving statistically significant results in primary end point.

^b^RCT: randomized controlled trial.

^c^Number of RCTs achieving statistically significant results in primary end point.

**Table 3:** Success rate of randomized controlled trials in achieving a reduction of glycated hemoglobin A_1c_ across the 3 intervention categories.

Intervention category	RCTs^a^ (n=23), n (%)	Significant results^b^, n/N (%)
High intensity	5 (22)	2/5 (40)
Medium intensity	14 (61)	8/14 (57)
Low intensity	4 (17)	2/4 (50)

^a^RCT: randomized controlled trial.

^b^Number of studies achieving statistically significant results in comparative reduction of HbA_1c_.

**Table 4:** Significant comparative reduction of glycated hemoglobin A_1c_ by intervention features.

Intervention feature			RCTs^a^ (n=23), n (%)		Significant results^b^, n/N (%)
**Communication mode**					
	Synchronous	12 (52)		4/12 (33)	
	Asynchronous	11 (48)		8/11 (73)	
**Frequency of communications**					
	Unlimited	7 (30)		4/7 (57)	
	Restricted	16 (70)		8/16 (50)	
**Qualification of coaches**					
	Diabetes specialists	11 (48)		6/11 (55)	
	General HCPs^c^	12 (52)		6/12 (50)	

^a^RCT: randomized controlled trial.

^b^Number of studies achieving statistically significant results in comparative reduction of glycated hemoglobin A_1c_.

^c^HCP: health care professional.

#### Study Quality Assessment and Risk of Bias

The Cochrane risk of bias tool [48] was used to assess the 23 RCTs (Multimedia Appendix 7). One (4%) study had a low risk of bias, 17 (74%) studies had some concerns regarding the overall risk of bias, and 5 (22%) studies had a high risk of bias overall (3 studies did not blind investigators, participants, or interventionists to group assignment [20,23,36]; 1 study used an inappropriate method of measuring HbA_1c_ (self-reported via a questionnaire) [26]; and 1 did not report how outcome data were collected) [37]. The Risk of Bias in Nonrandomized Studies–Interventions assessment tool [49] was used to evaluate the 5 nonrandomized interventional studies (Multimedia Appendix 8). Overall, 3 (60%) studies had a low risk of bias, and 2 (40%) studies were considered to contain insufficient information on which to base a judgment about the risk of bias, mainly because of missing information in one or more key domains [35,44].

### Meta-Analysis

#### Primary Analyses

In the primary analysis, the random effects meta-analysis (20 studies) [20-25,27,28,30,33,34,36-43,45] comparing the change in HbA_1c_ for intervention (number of patients across all studies, n*=*1637) versus usual care (n*=*1389) estimated an MD of –0.31% (95% CI –0.45% to –0.16%; *P*<.001). Heterogeneity was statistically significant (*Q*=57.64; *df*=19, *P*<.001), with an estimated τ of 0.21 (95% CI 0.12-0.61) and *I*^2^ of 67.54% (95% CI 41.19%-94.48%).

**Figure 2:**
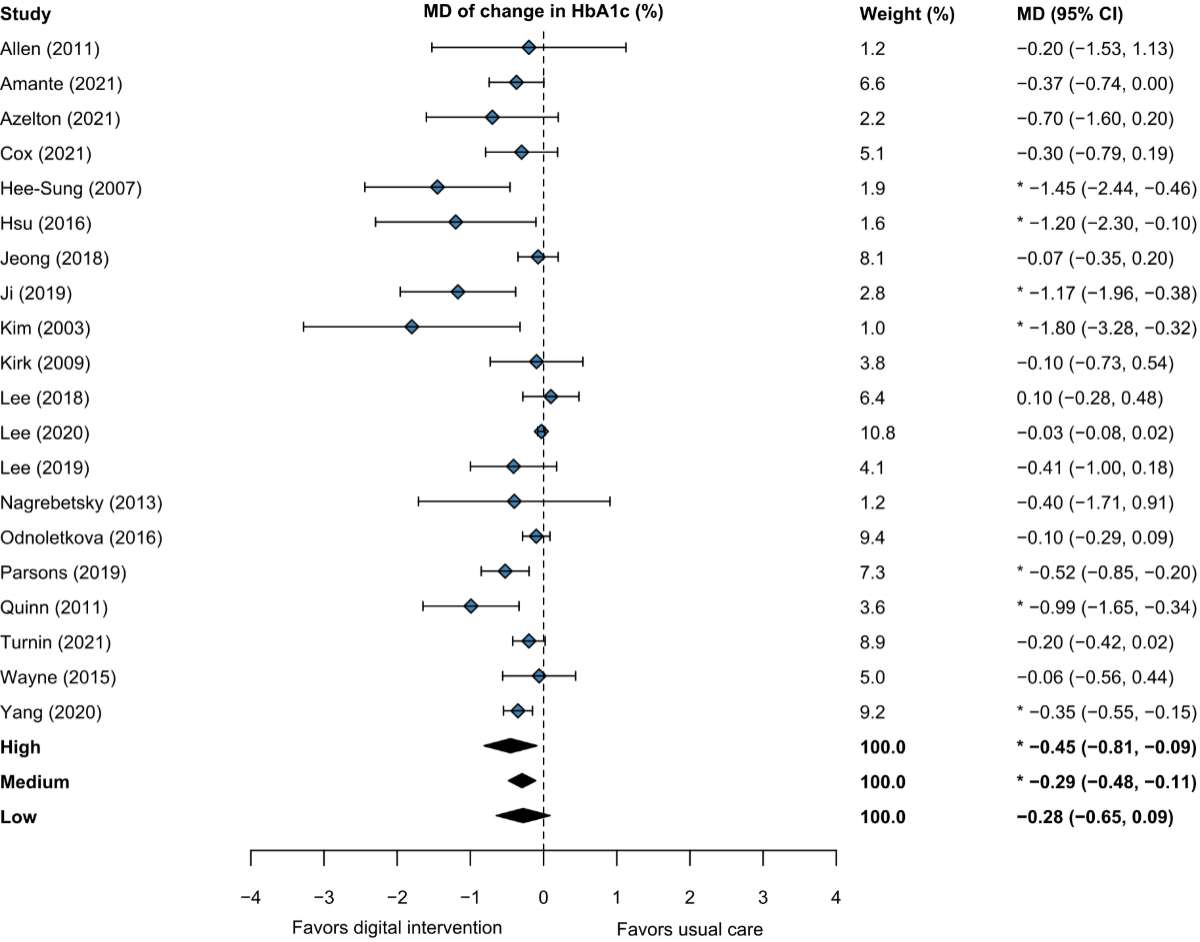
Forest plot of the MD and CI of change in HbA_1c_ with meta-regression on intervention intensity. * indicate statistical significance. HbA_1c_: glycated hemoglobin A_1c_; MD: mean difference.

In the meta-regression of studies that measured HbA_1c_ in a laboratory with intervention intensity as a categorical covariate (Figure 2), random effects meta-analysis (20 studies) [20-25,27,28,30,33,34,36-43,45] of change in HbA_1c_ on intensity for intervention (n*=*1637) versus usual care (n*=*1389) was estimated using the following equation:



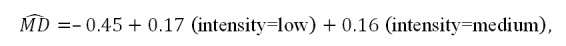



where 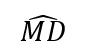 is the predicted MD, “intensity=low” is 1 if the intervention is low intensity and 0 otherwise; and “intensity=medium” is 1 if the intervention is medium intensity and 0 otherwise. This predicts an MD of –0.45% (95% CI –0.81% to –0.09%; *P*=.02) for high-intensity interventions, –0.29% (95% CI –0.48% to –0.11%; *P*=.003) for medium-intensity interventions, and –0.28% (95% CI –0.65% to 0.09%; *P*=.20) for low-intensity interventions. The low-intensity (*P*=.51) and medium-intensity (*P*=.45) coefficients were not statistically significant. Heterogeneity was statistically significant (*Q*=50.84, *df*=17, *P*<.001), with an estimated τ of 0.23 (95% CI 0.13-0.68) and *I*^2^ of 67.74% (95% CI 40.87%-94.72%).

#### Subgroup Analyses

For the high-intensity interventions, the random effects meta-analysis (4 studies) [20,22,41,45] of change in HbA_1c_ for intervention (n=253) versus control (n=201) estimated an MD of –0.46% (95% CI –0.84% to –0.07%; *P*<.02). Heterogeneity was statistically significant (*Q*=5.35, *df*=3, *P*<.20), with an estimated τ of 0.27 (95% CI 0.00-1.47) and *I^2^* of 46.77% (95% CI 0.00%-96.43%).

For the medium-intensity interventions, the random effects meta-analysis (12 studies) [21,23,25,27,30,33,37-40,42,43] of change in HbA_1c_ for intervention (n*=*989) versus control (n*=*847) estimated an MD of –0.28% (95% CI –0.45% to –0.11%; *P*<.002). Heterogeneity was statistically significant (*Q*=38.76, *df*=11, *P*<.001), with an estimated τ of 0.20 (95% CI 0.11-0.87) and *I*^2^ of 68.18% (95% CI 39.74%-97.57%).

For the combined high- and medium-intensity interventions, the random effects meta-analysis (16 studies) [20-23,25,27,30,33,36-43,45] of change in HbA_1c_ for intervention (n*=*1242) versus usual care (n*=*1048) estimated an MD of –0.32% (95% CI –0.47% to –0.16%; *P*<.001). Heterogeneity was statistically significant (*Q*=50.34, *df*=15, *P*<.001), with an estimated τ of 0.21 (95% CI 0.12-0.68) and *I*^2^ of 67.49% (95% CI 38.55%-95.46%).

For the low-intensity interventions, random effects meta-analysis (4 studies) [24,28,34,36] of change in HbA_1c_ for intervention (n=395) versus usual care (n=341) estimated an MD of –0.34% (95% CI –0.83 to 0.16; *P*<.2). Heterogeneity was not statistically significant (*P*<.09; *Q*=6.73, *df*=3), with an estimated τ of 0.38 (95% CI 0.00-1.90) and *I*^2^ of 60.64% (95% CI 0.00 to 97.50).

#### Sensitivity Analyses

Three sensitivity analyses were performed using (1) inclusion of studies using nonlaboratory-based HbA_1c_ measurement, (2) exclusion of studies that used CGM, and (3) exclusion of studies identified as high risk of bias according to the Cochrane risk of bias assessment. The results of all 3 analyses were in line with the primary analysis yielding a significant MD effect in favor of the digital intervention as compared with usual care (Table 5).

**Table 5:** Summary results of all conducted analyses.

Analysis set					Studies, n			Pooled mean difference estimate (%; 95% CI)			Significant heterogeneity
**Primary**											
	Studies with laboratory-measured HbA_1c_^a^			20			–0.31 (–0.45 to –0.16)^b^			Yes	
	**Studies with laboratory-measured HbA_1c_, meta-regression**										Yes
		High	20			–0.45 (–0.81 to –0.09)^b^					
		Medium	20			–0.29% (–0.48 to –0.11)^b^					
		Low	20			–0.28% (–0.65 to 0.09)^c^					
**Subgroups**											
	High-intensity interventions			4			–0.43 (–0.78 to –0.09)^b^			No	
	Medium-intensity interventions			12			–0.28 (–0.45 to, –0.11)^b^			Yes	
	High- and medium-intensity interventions			16			–0.32 (–0.47 to –0.16)^b^			Yes	
	Low-intensity interventions			4			–0.34 (–0.83 to 0.16)^c^			No	
**Sensitivity**											
	Including nonlaboratory-measured HbA_1c_ studies			23			–0.40 (–0.56 to –0.24)^b^			Yes	
	Excluding continuous glucose monitoring studies			18			–0.31 (–0.47 to –0.15)^b^			Yes	
	Excluding high-risk studies			16			–0.31 (–0.46 to –0.15)^b^			Yes	

^a^HbA_1c_: glycated hemoglobin A_1c_.

^b^Statistically significant.

^c^Nonsignificant.

## Discussion

### Principal Findings

In this SLR and meta-analysis, the currently available evidence suggests that the use of digital health interventions, compared with usual care, is associated with clinically significant improvement in HbA_1c_ levels for individuals with T2DM. Furthermore, the intensity of support provided by HCPs also appears to impact the HbA_1c_ levels. Here, intensity included the types and frequency of interactions between professionals and people with T2DM as well as the qualifications of the professional.

Although most (17/23, 74%) of the RCTs reported their primary end points as defined by the study protocol, achieving a significant comparative reduction of HbA_1c_ between digital health intervention and usual care appears to be challenging. Only half (12/23, 52%) of the RCTs reported that the digital health interventions, compared with usual care, were successful in achieving a statistically significant difference in HbA_1c_ reduction. In addition, there was variability in the performance of different digital interventions. Based on this analysis, 2 essential components of each intervention—technology and coaching—seem to independently influence the outcomes. Information about the self-measured glucose levels was available to participants in all included studies. With 1 exception, the intervention arm included either self-monitoring of blood glucose or CGM devices. The devices provided on-demand glucose data to the person using the device as well as to the supervising health care team. Therefore, the availability of raw glucose data does not account for the observed outcome differences between the studies since the glucose data were available to both the intervention group and the control group in all studies. Even the presence or absence of additional devices (such as connected weight scales [46] or accelerometers [25,34]) did not appear to make a difference. Consequently, easy access to self-measured data alone did not seem to be a sufficient condition for improved glycemic control.

In addition to the data provided by the devices, the other aspect of the intervention was coaching. Coaching can be stratified into 2 components: education and counseling. All studies provided educational sessions to the participants, albeit the extent and quality of the education varied. Some studies provided only basic forms of education usually based on preexisting materials published by outside sources (such as the American Diabetes Association guidelines [37,45] or Diabetes UK [34]). The educational content was restricted to general diabetes information and to the technical aspects of blood glucose monitoring [24,27,30,38]. The educational sessions were led by nurses and the participants were mostly receiving preprinted materials and watching prerecorded videos. In higher-tier interventions (as defined in the *Methods* section), the educational materials were usually produced in house by the institution conducting the study [20,29,35,36] and tailored to the needs of the target population. The educational sessions were led by specialists (such as diabetes educators [20,36], dietitians [29,31], or pharmacists [38]) and often in small groups or in one-on-one settings. The sessions had the active participation of both the health care staff and the participants.

The coaching element was the most distinctive feature which differentiated the more intensive interventions from the lower-tier interventions (Table 2). The top-tier interventions provided regular, individualized coaching sessions with trained diabetes educators, using graphical visualization tools to go over an individual’s data with them and advise on the best course of action. Sessions were in person [36] or remote via videoconferencing [35,41,45], voice call [26], SMS text messaging [46], or a mixture of these. The content of the coaching sessions was tailored to the specific goals of the digital intervention. Three examples of such coaching are problem-solving [36], where participants were asked to discuss their specific barriers in implementing the intervention; development of an individualized plan to improve problem-solving skills and self-care [41], implemented within the diabetes program and targeting 7 self-care behaviors; and motivational interviewing, goal-setting, and confidence building [20].

Across the high-intensity studies (including the 3 examples listed earlier), coaching was frequent, available on-demand, and tailored for the individual. The medium for communication did not appear to be important.

Further, additional features of the interventions such as mode of communication, frequency of the communications, and qualification of the coaches were also important. The most pronounced difference was between studies using synchronous compared with asynchronous communication, with the advantage favoring the use of an asynchronous mode of communication. This finding appears counterintuitive, as direct human-to-human contact is the most common way of coaching (health or otherwise). One possible explanation may come from the fact that the unlimited frequency of communication also seems to have a slight advantage over restricted or scheduled communications. The availability of the coaches for direct contact is constrained by the patient-to-coach ratios and the limited amount of time that each coach will be available in real time. On the other hand, asynchronous communication modalities such as text messages and emails allow for near-real-time communication without the logistic constraints of direct interactions.

This finding is novel and not yet supported by other studies. A systematic review of longitudinal management of chronic conditions by telehealth interventions [8] reported no difference between asynchronous and in-person (synchronous) reviews of patient data. However, the asynchronous mode was represented by a dedicated web page rather than text messaging, so the comparison may not be quite relevant. In an umbrella review of technology-enabled diabetes self-management [50], a new taxonomy for digital interventions was proposed. This taxonomy includes a distinction between synchronous and asynchronous feedback modes, but the authors noted poor reporting on this issue in the reviewed studies. Here, the qualifications of the coaches were the least significant factor in this analysis. However, all included RCTs were driven by a protocol outlining the important details of the intervention. All staff participating in those interventions were therefore instructed before the beginning of the trial in the proper method of coaching and patient interactions. Even the nondiabetes specialists were given specialized instructions on how to approach the patients which may have contributed to blurring the distinction between diabetes specialists and general HCPs.

When analyzing the comparative reduction of HbA_1c_ within the context of RCTs, the meta-analysis confirmed the findings from the SLR. A statistically significant reduction in HbA_1c_ relative to usual care was observed globally for high- and medium-intensity interventions, but not for low-intensity interventions. Meta-regression coefficients were not statistically significant, and hence no support was found for a difference in efficacy according to intervention intensity, but this finding was limited by the relatively small number of high- and low-intensity interventions. The relatively modest, although statistically significant, effect size observed in the comparative reduction of HbA_1c_ levels may be explained by several factors present in all included RCTs. First, the participants in the comparator arms of the studies were receiving usual diabetes care and the results show that this level of care also reduced their HbA_1c_. In this context, digital interventions can be viewed as an adjunct therapy. Second, the improved efficacy relative to the usual care might not be the only advantage of digital interventions. Finally, the effect size observed in this meta-analysis is similar to the one reported elsewhere investigating mobile health efficacy in diabetes treatment and management across developing and developed countries [51]. In addition, digital intervention can induce behavior change via coaching sessions that include problem-solving and identifying barriers, so the effects of HbA_1c_ improvement caused by this behavior change could be sustainable compared with usual care.

The sensitivity analysis including studies with non–laboratory-measured HbA_1c_ levels resulted in a more favorable result for digital interventions. This suggests that the decision to exclude those studies led to a more conservative result. The other 2 sensitivity analyses did not change the result, and so we conclude that the inclusion of CGM and high-risk of bias studies was not a determining factor in the results. One caveat to the observation that the inclusion of CGM did not change the results was the fact that the number of studies with CGM was 2. In this analysis, the evidence base had studies spanning a broad range of years including early years when CGM was less prevalent.

In the studies from this review investigating engagement, the results showed that the digital intervention led to a significant difference between the intervention and usual care groups in terms of changes in self-care behaviors, and that digital interventions are well received with high completion rates and no additional burden. In the 3 studies investigating patient satisfaction using DTSQ questionnaires, all reported significant improvement in DTSQ scores in the digital intervention groups, suggesting a high satisfaction with the treatment. A challenge facing the reports on engagement is the lack of clear differentiation between patient participant adherence and engagement. Those 2 concepts are often used interchangeably; however, they refer to different aspects of an individual’s behavior [52]. The usual definition of patient adherence includes a willingness to follow the study protocol in all aspects. Engagement includes an individual’s initiative to actively seek improvement in their disease management. This distinction is important for differentiating between passive following of instructions and self-initiated activities.

A total of 11 (39%) of the 28 included studies reported on adverse events. Seven of these studies reported no intervention-related adverse events and 4 studies did report the occurrence of adverse events without stating whether those were related to the intervention or not. Given the nature of these events and the fact that the participants remained on their previously prescribed medication regimen, the link between the digital interventions and the adverse events is difficult to establish. Overall, the reporting on adverse events in the included studies was poor and this constitutes an unmet need in the domain of digital interventions.

### Limitations of the Study

There are multiple limitations to our review and meta-analysis. First, the apparent relationships and conclusions regarding the intensity of the digital intervention must be tested in a prospective manner to see whether they prove to be valid. Next, the heterogeneous nature of the featured interventions makes it difficult to generalize the findings. This has been a consistent theme and conclusion in other systematic reviews with or without meta-analyses in digital health. The heterogeneity can be seen in a variety of study settings across multiple countries with different cultures that may influence the acceptance of the intervention. This aspect was not addressed in the individual studies. Our approach in this study was to stratify the interventions based on the intensity of the coaching as the most distinctive pattern among the interventions. However, because of a wide variety of coaching strategies, clear boundaries between the categories were not easy to draw. This was also true for selecting the 3 additional features of the interventions (communication mode, frequency of communications, and qualification of the coaches) used to further investigate the factors contributing to efficacy.

The meta-regression and subgroup analyses were limited by the small number of high- and low-intensity interventions, which resulted in low power to detect differences in HbA_1c_ reduction according to intervention intensity. The effect of time of follow-up was also not investigated.

As well, only RCTs were included in the meta-analysis. Although this reduced the introduction of bias associated with nonrandomized studies, it does limit the generalizability of the findings in the real world [53]. The risk-of-bias analysis revealed that all RCTs in the evidence base contained some degree of bias. Although no difference in the degree of bias between 1 intervention and another was found, the presence of bias confounds the results of the meta-analysis.

With respect to the scalability of the interventions in the real world, the need for the dedicated staff of HCPs to support the higher-intensity studies adds additional economic and logistic burden. Some of the solutions may require a dedicated database; communication infrastructure; customized user software; and trained, professional staff. After adding the necessary maintenance expenditures, the overall cost of these solutions may be out of the reach of certain clinics. Finally, some digital interventions are intended to deliver behavior change, but there was a paucity of clear evidence that behavior changed because the behavior change aspects of the intervention were not measured appropriately.

### Recommendations for Future Research

Based on the findings, recommendations for future research in digital health include the following. (1) An agreed definition of engagement in digital health as an end point may help with improved targeting of interventions. (2) Reporting should standardize digital health data into meaningful outcomes by therapeutic area (and then beyond), such as sensor data and patient-reported outcome measures, so that future systematic reviews and meta-analyses can be less heterogeneous. (3) Studies in digital interventions should strive for a clear reporting of adverse events, especially in terms of the relationship between the digital health product and the adverse events. (4) Digital health studies that include coaching should systematically record multiple dimensions of the intervention, including frequency, duration, asynchronous versus synchronous, coaching or behavior change techniques deployed, human coach qualification (if relevant), and guidance and introduction to patients. For methodological purposes, a newly developed scoring system for the classification of the intensity of coaching would help future analyses of digital interventions. (5) Consider different designs and methodologies to study digital health interventions, especially those that are intended to deliver behavior change, so that meaningful patient engagement in the digital solution and outcome measures aligned with intended use can be assessed. To eliminate bias, perhaps cluster randomization (or some other method for eliminating bias) should be used in future digital health interventions.

### Conclusions

Reducing HbA_1c_ levels in patients with T2DM with the use of digital interventions, in addition to usual care, is feasible and acceptable to people with T2DM, as consistently demonstrated by a large number of studies of various populations, goals, and methods of interventions. When analyzing the comparative efficacy of digital interventions within the context of RCTs, the advantage of digital interventions becomes less pronounced. Some forms of intervention perform better than others, but it is difficult to identify the exact reasons for this difference given the variety of methodologies featured in the studies. However, a broadly defined intensity of coaching seems to play an important role. A common feature of successful studies was the availability of timely and responsive personalized coaching. Therefore, the relevance and the content of the coaching are more important than the communication medium used to deliver the messages. Scaling up the personalized, on-demand coaching featured in some of the studies may lead to logistical and economic roadblocks. Overcoming these roadblocks will largely determine the success of digital interventions in real-world clinical practice. In conclusion, digital health interventions for diabetes appear to be a useful tool for improving outcomes.

## Appendix

Multimedia Appendix 1 PICO (Population, Intervention, Comparator, and Outcomes) criteria used for study selection.

Multimedia Appendix 2 Search strategy for Embase via OvidSP.

Multimedia Appendix 3 Search strategy for MEDLINE via OvidSP.

Multimedia Appendix 4 Search strategy for CENTRAL via OvidSP.

Multimedia Appendix 5 Distribution of the mean baseline HbA_1c_ levels across included studies reporting. HbA_1c_: glycated hemoglobin A_1c_.

Multimedia Appendix 6 Categorization of the studies into the intervention categories.

Multimedia Appendix 7 Risk of bias as percentage across randomized controlled trials (intention to treat).

Multimedia Appendix 8 Risk of bias in nonrandomized studies.

## Abbreviations

CGM: continuous glucose monitoring
DTSQ: Diabetes Treatment Satisfaction Questionnaire
HbA_1c_: glycated hemoglobin A_1c_
HCP: health care professional
MD: mean difference
PICO: Population, Intervention, Comparator, and Outcomes
PRISMA: Preferred Reporting Items for Systematic Reviews and Meta-Analyses
RCT: randomized controlled trial
SLR: systematic literature review
T2DM: type 2 diabetes mellitus

## Data Availability

The data sets generated and analyzed during this study are available from the corresponding author on reasonable request.

## References

[1] Silberman J, Wicks P, Patel S, Sarlati S, Park S, Korolev IO, Carl JR, Owusu JT, Mishra V, Kaur M, Willey VJ, Sucala ML, Campellone TR, Geoghegan C, Rodriguez-Chavez IR, Vandendriessche B (2023). Rigorous and rapid evidence assessment in digital health with the evidence DEFINED framework. NPJ Digit Med.

[2] Fontecha J, González I, Barragán A, Lim T (2022). Use and trends of diabetes self-management technologies: a correlation-based study. J Diabetes Res.

[3] Allegrante JP, Wells MT, Peterson JC (2019). Interventions to support behavioral self-management of chronic diseases. Annu Rev Public Health.

[4] Pal K, Dack C, Ross J, Michie S, May C, Stevenson F, Farmer A, Yardley L, Barnard M, Murray E (2018). Digital health interventions for adults with type 2 diabetes: qualitative study of patient perspectives on diabetes self-management education and support. J Med Internet Res.

[5] Jarl F, Davelid A, Hedin K, Stomby A, Petersson C (2023). Overcoming the struggle of living with type 2 diabetes – diabetes specialist nurses' and patients' perspectives on digital interventions. BMC Health Serv Res.

[6] Bults M, van Leersum CM, Olthuis TJJ, Bekhuis REM, den Ouden MEM (2023). Mobile health apps for the control and self-management of type 2 diabetes mellitus: qualitative study on users' acceptability and acceptance. JMIR Diabetes.

[7] ElSayed NA, Aleppo G, Aroda VR, Bannuru RR, Brown FM, Bruemmer D, Collins BS, Hilliard ME, Isaacs D, Johnson EL, Kahan S, Khunti K, Leon J, Lyons SK, Perry ML, Prahalad P, Pratley RE, Seley JJ, Stanton RC, Gabbay RA (2023). 7. Diabetes technology: standards of care in diabetes–2023. Diabetes Care.

[8] Lewinski AA, Walsh C, Rushton S, Soliman D, Carlson SM, Luedke MW, Halpern DJ, Crowley MJ, Shaw RJ, Sharpe JA, Alexopoulos A, Tabriz AA, Dietch JR, Uthappa DM, Hwang S, Ball Ricks KA, Cantrell S, Kosinski AS, Ear B, Gordon AM, Gierisch JM, Williams JW, Goldstein KM (2022). Telehealth for the longitudinal management of chronic conditions: systematic review. J Med Internet Res.

[9] ElSayed NA, Aleppo G, Aroda VR, Bannuru RR, Brown FM, Bruemmer D, Collins BS, Hilliard ME, Isaacs D, Johnson EL, Kahan S, Khunti K, Leon J, Lyons SK, Perry ML, Prahalad P, Pratley RE, Seley JJ, Stanton RC, Gabbay RA (2023). 6. Glycemic targets: standards of care in diabetes–2023. Diabetes Care.

[10] Bin Rakhis SA, AlDuwayhis NM, Aleid N, AlBarrak AN, Aloraini AA (2022). Glycemic control for type 2 diabetes mellitus patients: a systematic review. Cureus.

[11] Eberle C, Löhnert Maxine, Stichling S (2021). Effectiveness of disease-specific mhealth apps in patients with diabetes mellitus: scoping review. JMIR Mhealth Uhealth.

[12] Eberle C, Stichling S (2021). Effect of telemetric interventions on glycated hemoglobin a1c and management of type 2 diabetes mellitus: systematic meta-review. J Med Internet Res.

[13] Kerr D, Messing R, Resch A (2011). Actionable self-monitoring of blood glucose: redefining the role for patients using multiple daily injection therapy. J Diabetes Sci Technol.

[14] Fu HNC, Wyman JF, Peden-McAlpine CJ, Draucker CB, Schleyer T, Adam TJ (2023). App design features important for diabetes self-management as determined by the self-determination theory on motivation: content analysis of survey responses from adults requiring insulin therapy. JMIR Diabetes.

[15] Fundoiano-Hershcovitz Y, Hirsch A, Dar S, Feniger E, Goldstein P (2021). Role of digital engagement in diabetes care beyond measurement: retrospective cohort study. JMIR Diabetes.

[16] ElSayed NA, Aleppo G, Aroda VR, Bannuru RR, Brown FM, Bruemmer D, Collins BS, Hilliard ME, Isaacs D, Johnson EL, Kahan S, Khunti K, Leon J, Lyons SK, Perry ML, Prahalad P, Pratley RE, Seley JJ, Stanton RC, Young-Hyman D, Gabbay RA (2023). 5. Facilitating positive health behaviors and well-being to improve health outcomes: standards of care in diabetes–2023. Diabetes Care.

[17] Hessler D, Strycker L, Fisher L (2021). Reductions in management distress following a randomized distress intervention are associated with improved diabetes behavioral and glycemic outcomes over time. Diabetes Care.

[18] Higgins JT, Chandler J, Cumpston M, Li T, Page M, Welch V (2019). Cochrane Handbook for Systematic Reviews of Interventions. 2nd Edition.

[19] Page MJ, McKenzie JE, Bossuyt PM, Boutron I, Hoffmann TC, Mulrow CD, Shamseer L, Tetzlaff JM, Akl EA, Brennan SE, Chou R, Glanville J, Grimshaw JM, Hróbjartsson A, Lalu MM, Li T, Loder EW, Mayo-Wilson E, McDonald S, McGuinness LA, Stewart LA, Thomas J, Tricco AC, Welch VA, Whiting P, Moher D (2021). The PRISMA 2020 statement: an updated guideline for reporting systematic reviews. BMJ.

[20] Azelton KR, Crowley AP, Vence N, Underwood K, Morris G, Kelly J, Landry MJ (2021). Digital health coaching for type 2 diabetes: randomized controlled trial of healthy at home. Front Digit Health.

[21] Turnin M, Gourdy P, Martini J, Buisson J, Chauchard M, Delaunay J, Schirr-Bonnans S, Taoui S, Poncet M, Cosma V, Lablanche S, Coustols-Valat M, Chaillous L, Thivolet C, Sanz C, Penfornis A, Lepage B, Colineaux H, Mounié M, Costa N, Molinier L, Hanaire H (2021). Impact of a remote monitoring programme including lifestyle education software in type 2 diabetes: results of the Educ@dom randomised multicentre study. Diabetes Ther.

[22] Wayne N, Perez DF, Kaplan DM, Ritvo P (2015). Health coaching reduces HbA1c in type 2 diabetic patients from a lower-socioeconomic status community: a randomized controlled trial. J Med Internet Res.

[23] Kim HS, Oh JA (2003). Adherence to diabetes control recommendations: impact of nurse telephone calls. J Adv Nurs.

[24] Odnoletkova I, Goderis G, Nobels F, Fieuws S, Aertgeerts B, Annemans L, Ramaekers D (2016). Optimizing diabetes control in people with type 2 diabetes through nurse-led telecoaching. Diabet Med.

[25] Cox DJ, Oser T, Moncrief M, Conaway M, McCall A (2021). Long-term follow-up of a randomized clinical trial comparing glycemic excursion minimization (GEM) to weight loss (WL) in the management of type 2 diabetes. BMJ Open Diabetes Res Care.

[26] Guo M, Meng F, Guo Q, Bai T, Hong Y, Song F, Ma Y (2023). Effectiveness of mHealth management with an implantable glucose sensor and a mobile application among Chinese adults with type 2 diabetes. J Telemed Telecare.

[27] Parsons SN, Luzio SD, Harvey JN, Bain SC, Cheung WY, Watkins A, Owens DR (2019). Effect of structured self-monitoring of blood glucose, with and without additional TeleCare support, on overall glycaemic control in non-insulin treated type 2 diabetes: the SMBG study, a 12-month randomized controlled trial. Diabet Med.

[28] Ji H, Chen R, Huang Y, Li W, Shi C, Zhou J (2019). Effect of simulation education and case management on glycemic control in type 2 diabetes. Diabetes Metab Res Rev.

[29] Pimazoni-Netto A, Rodbard D, Zanella MT, Diabetes EducationControl Group (2011). Rapid improvement of glycemic control in type 2 diabetes using weekly intensive multifactorial interventions: structured glucose monitoring, patient education, and adjustment of therapy–a randomized controlled trial. Diabetes Technol Ther.

[30] Lee DY, Park J, Choi D, Ahn H, Park S, Park C (2018). The effectiveness, reproducibility, and durability of tailored mobile coaching on diabetes management in policyholders: a randomized, controlled, open-label study. Sci Rep.

[31] Athinarayanan SJ, Adams RN, Hallberg SJ, McKenzie AL, Bhanpuri NH, Campbell WW, Volek JS, Phinney SD, McCarter JP (2019). Long-term effects of a novel continuous remote care intervention including nutritional ketosis for the management of type 2 diabetes: a 2-year non-randomized clinical trial. Front Endocrinol.

[32] Welch G, Zagarins SE, Santiago-Kelly P, Rodriguez Z, Bursell SE, Rosal MC, Gabbay RA (2015). An internet-based diabetes management platform improves team care and outcomes in an urban Latino population. Diabetes Care.

[33] Yang Y, Lee EY, Kim HS, Lee SH, Yoon KH, Cho JH (2020). Effect of a mobile phone–based glucose-monitoring and feedback system for type 2 diabetes management in multiple primary care clinic settings: cluster randomized controlled trial. JMIR Mhealth Uhealth.

[34] Kirk A, Barnett J, Leese G, Mutrie N (2009). A randomized trial investigating the 12-month changes in physical activity and health outcomes following a physical activity consultation delivered by a person or in written form in type 2 diabetes: Time2Act. Diabet Med.

[35] Layne JE, Bergenstal RM, Barleen NA, Dixon RF, Zisser H (2021). 597-P: long-term A1C outcomes with and without intermittent CGM use in adults with T2D participating in the Onduo program. Diabetes.

[36] Allen N, Whittemore R, Melkus G (2011). A continuous glucose monitoring and problem-solving intervention to change physical activity behavior in women with type 2 diabetes: a pilot study. Diabetes Technol Ther.

[37] Jeong JY, Jeon JH, Bae KH, Choi YK, Park KG, Kim JG, Won KC, Cha BS, Ahn CW, Kim DW, Lee CH, Lee IK (2018). Smart care based on telemonitoring and telemedicine for type 2 diabetes care: multi-center randomized controlled trial. Telemed J E Health.

[38] Lee JY, Chan CKY, Chua SS, Ng CJ, Paraidathathu T, Lee KKC, Lee SWH (2020). Telemonitoring and team-based management of glycemic control on people with type 2 diabetes: a cluster-randomized controlled trial. J Gen Intern Med.

[39] Hsu WC, Lau KHK, Huang R, Ghiloni S, Le H, Gilroy S, Abrahamson M, Moore J (2016). Utilization of a cloud-based diabetes management program for insulin initiation and titration enables collaborative decision making between healthcare providers and patients. Diabetes Technol Ther.

[40] Hee-Sung Kim (2007). Impact of web-based nurse's education on glycosylated haemoglobin in type 2 diabetic patients. J Clin Nurs.

[41] Amante DJ, Harlan DM, Lemon SC, McManus DD, Olaitan OO, Pagoto SL, Gerber BS, Thompson MJ (2021). Evaluation of a diabetes remote monitoring program facilitated by connected glucose meters for patients with poorly controlled type 2 diabetes: randomized crossover trial. JMIR Diabetes.

[42] Nagrebetsky A, Larsen M, Craven A, Turner J, McRobert N, Murray E, Gibson O, Neil A, Tarassenko L, Farmer A (2013). Stepwise self-titration of oral glucose-lowering medication using a mobile telephone-based telehealth platform in type 2 diabetes: a feasibility trial in primary care. J Diabetes Sci Technol.

[43] Lee SK, Shin DH, Kim YH, Lee KS (2019). Effect of diabetes education through pattern management on self-care and self-efficacy in patients with type 2 diabetes. Int J Environ Res Public Health.

[44] Das S, Manikandan R, Boochandran T, Ahmed A, Dwarakanath C, Jaganmohan B (2018). Comprehensive Apollo sugar diabetes management programs: adherence for better clinical outcomes. Indian J Endocrinol Metab.

[45] Quinn CC, Shardell MD, Terrin ML, Barr EA, Ballew SH, Gruber-Baldini AL (2011). Cluster-randomized trial of a mobile phone personalized behavioral intervention for blood glucose control. Diabetes Care.

[46] McKenzie AL, Hallberg SJ, Creighton BC, Volk BM, Link TM, Abner MK, Glon RM, McCarter JP, Volek JS, Phinney SD (2017). A novel intervention including individualized nutritional recommendations reduces hemoglobin A1c level, medication use, and weight in type 2 diabetes. JMIR Diabetes.

[47] Fantasia KL, Stockman MC, Ju Z, Ortega P, Crable EL, Drainoni ML, Walkey AJ, Bergstrom M, O'Brien K, Steenkamp D (2021). Professional continuous glucose monitoring and endocrinology econsult for adults with type 2 diabetes in primary care: results of a clinical pilot program. J Clin Transl Endocrinol.

[48] Sterne JAC, Savović J, Page MJ, Elbers RG, Blencowe NS, Boutron I, Cates CJ, Cheng H, Corbett MS, Eldridge SM, Emberson JR, Hernán MA, Hopewell S, Hróbjartsson A, Junqueira DR, Jüni P, Kirkham JJ, Lasserson T, Li T, McAleenan A, Reeves BC, Shepperd S, Shrier I, Stewart LA, Tilling K, White IR, Whiting PF, Higgins JPT (2019). RoB 2: a revised tool for assessing risk of bias in randomised trials. BMJ.

[49] Sterne JA, Hernán MA, Reeves BC, Savović J, Berkman ND, Viswanathan M, Henry D, Altman DG, Ansari MT, Boutron I, Carpenter JR, Chan A, Churchill R, Deeks JJ, Hróbjartsson A, Kirkham J, Jüni P, Loke YK, Pigott TD, Ramsay CR, Regidor D, Rothstein HR, Sandhu L, Santaguida PL, Schünemann HJ, Shea B, Shrier I, Tugwell P, Turner L, Valentine JC, Waddington H, Waters E, Wells GA, Whiting PF, Higgins JP (2016). ROBINS-I: a tool for assessing risk of bias in non-randomised studies of interventions. BMJ.

[50] Greenwood DA, Litchman ML, Isaacs D, Blanchette JE, Dickinson JK, Hughes A, Colicchio VD, Ye J, Yehl K, Todd A, Peeples MM (2022). A new taxonomy for technology-enabled diabetes self-management interventions: results of an umbrella review. J Diabetes Sci Technol.

[51] Mao Y, Lin W, Wen J, Chen G (2020). Impact and efficacy of mobile health intervention in the management of diabetes and hypertension: a systematic review and meta-analysis. BMJ Open Diabetes Res Care.

[52] Marzban S, Najafi M, Agolli A, Ashrafi E (2022). Impact of patient engagement on healthcare quality: a scoping review. J Patient Exp.

[53] Klonoff DC, Gutierrez A, Fleming A, Kerr D (2019). Real-world evidence should be used in regulatory decisions about new pharmaceutical and medical device products for diabetes. J Diabetes Sci Technol.

