# Supplementary material for: Digital Interventions for Self-Management of Type 2 Diabetes Mellitus: Systematic Literature Review and Meta-Analysis
Source: J Med Internet Res. 2024 Jul 22;26:e55757. doi: 10.2196/55757 (PMC11301119; doi:10.2196/55757)
Supplement: Multimedia Appendix 1 [file jmir_v26i1e55757_app1.docx]

**Multimedia Appendix 1**

| **PICO item** | **Inclusion criteria** | **Exclusion criteria** |
| --- | --- | --- |
| **Population** | Adults (>18 years of age) with type 2 diabetes mellitus | - Children - Adolescents |
| **Interventions** | Integrated solutions containing both human and digital components:   - Digital component, including but not limited to patient data capture devices:   - Glucose meter (required)   - Blood pressure cuff   - Digital scale   - Biofeedback sensors - Human component:   Remote, or hybrid remote/in-person, coaching by healthcare professional | - Fully digital solutions with no human component - Minimal digital solutions |
| **Comparators** | Any or none | - N/A |
| **Outcomes** | - Primary outcome   - HbA1c estimates (focus for timepoints: ≥3 months) - Secondary outcome - Engagement rates (e.g., user engagement, retention, withdrawal) | - N/A |
| **Study design** | - Randomized controlled trials - Quasi-randomized trials with control group - Observational studies using matching techniques - Observational studies with a control group | - Non-comparative studies (e.g., single-arm trials) - Case series, case studies - Reviews - Meta-analyses |
| **Additional criteria (limits)** | | |
| **Language** | English | |

N/A = not applicable
