# Supplementary material for: Digital Interventions for Self-Management of Type 2 Diabetes Mellitus: Systematic Literature Review and Meta-Analysis
Source: J Med Internet Res. 2024 Jul 22;26:e55757. doi: 10.2196/55757 (PMC11301119; doi:10.2196/55757)
Supplement: Multimedia Appendix 4 [file jmir_v26i1e55757_app4.docx]

**Multimedia Appendix 4**

| **Database: EBM Reviews - Cochrane Central Register of Controlled Trials <January 2022>**  **Search executed: April 5, 2022** | | |
| --- | --- | --- |
| **#** | **String** | **Hits** |
| 1 | exp Diabetes Mellitus, Type 2/ | 19443 |
| 2 | (("type 2" or "type II" or "type two" or adult or (ketosis adj resistan*) or matur* or late or "noninsulin" or "non-insulin" or slow* or stable or lipoatrophic) adj2 (diabete* or diabetic*)).ti,ab. | 44355 |
| 3 | ("Mody" or "niddm" or "t2dm").ti,ab. | 8530 |
| 4 | or/1-3 | 47808 |
| 5 | (("hybrid" or "in-person" or "inperson" or "virtual" or "digital" or "platform" or "remote" or "tele" or "mobile" or "smartphone" or "smart phone" or "mobile phone" or "mobilephone" or "cellular") adj2 ("coaching" or consult* or guidance* or "guiding" or train* or workshop* or instructor* or tutor* or educator* or education*)).ti,ab. | 3513 |
| 6 | (("virtual" or "digital" or "platform" or "remote" or "tele") adj2 (management* or "system" or therapeutic* or "solutions" or "monitoring")).ti,ab. | 2484 |
| 7 | (("cellular" or "smart" or "remote" or "connected" or "flash") adj2 ("hba1c" or "Hemoglobin A1C" or "glucose" or "glycated haemoglobin" or "glycated hemoglobin")).ti,ab. | 343 |
| 8 | ("self-measurement of blood glucose" or "Self-Monitoring of Blood Glucose" or "SMBG" or "remote monitoring" or "smart glucose meter" or "continuous glucose monitoring" or "CGM" or "flash glucose monitoring").ti,ab. | 4607 |
| 9 | ("dario" or "dariohealth" or "vida" or "livongo" or "omada" or "lark" or "noom" or "onduo" or "onedrop" or "one drop" or "welldoc" or "virta").ti,ab. | 1475 |
| 10 | or/5-9 | 11139 |
| 11 | 4 and 10 | 1908 |
| 12 | limit 11 to english | 1154 |
