# Supplementary figures and images for: Digital Interventions for Self-Management of Type 2 Diabetes Mellitus: Systematic Literature Review and Meta-Analysis
Source: J Med Internet Res. 2024 Jul 22;26:e55757. doi: 10.2196/55757 (PMC11301119; doi:10.2196/55757)

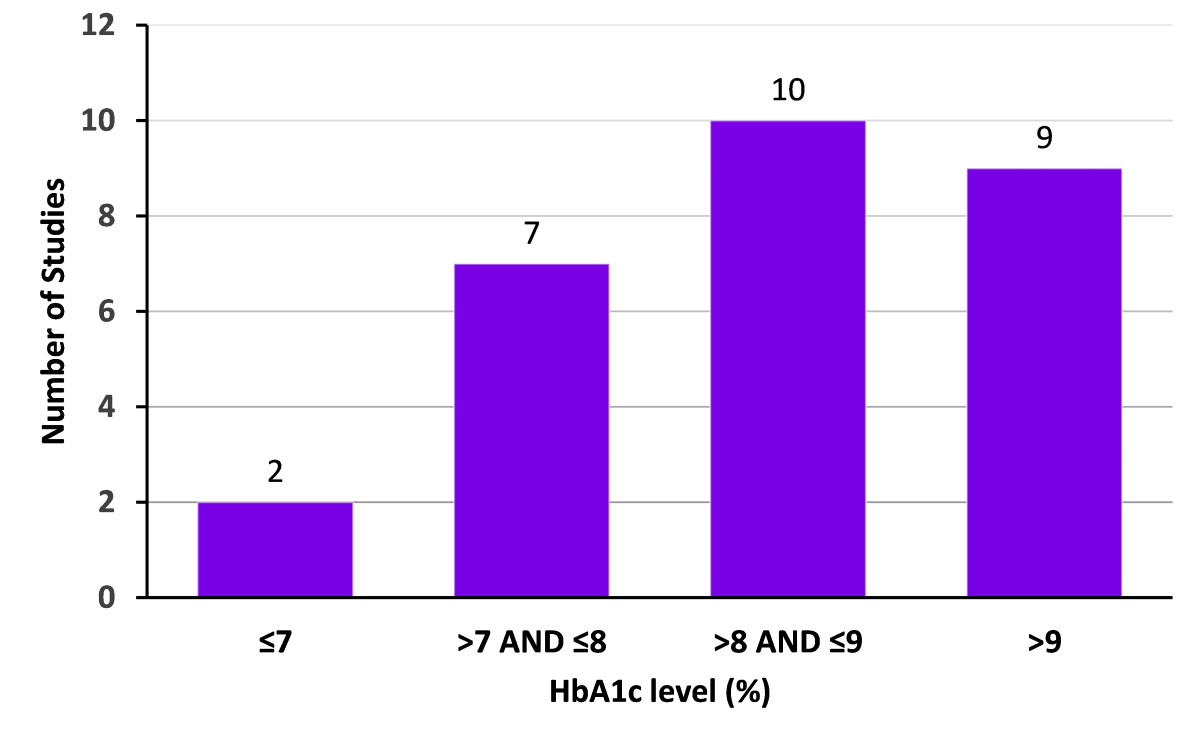

Supplement: Multimedia Appendix 5 [file jmir_v26i1e55757_app5.png]

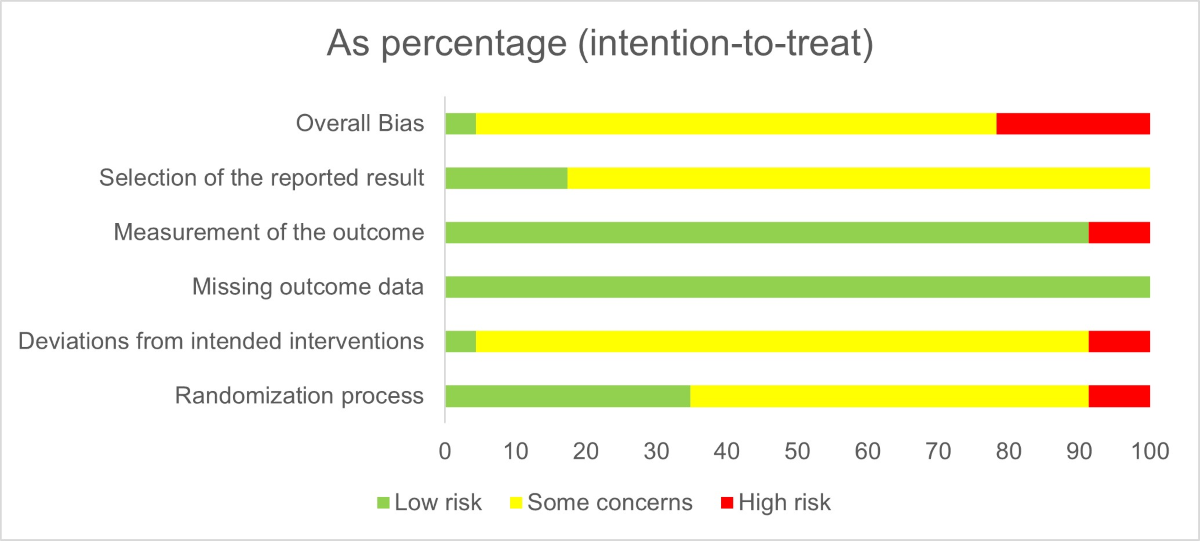

Supplement: Multimedia Appendix 7 [file jmir_v26i1e55757_app7.png]

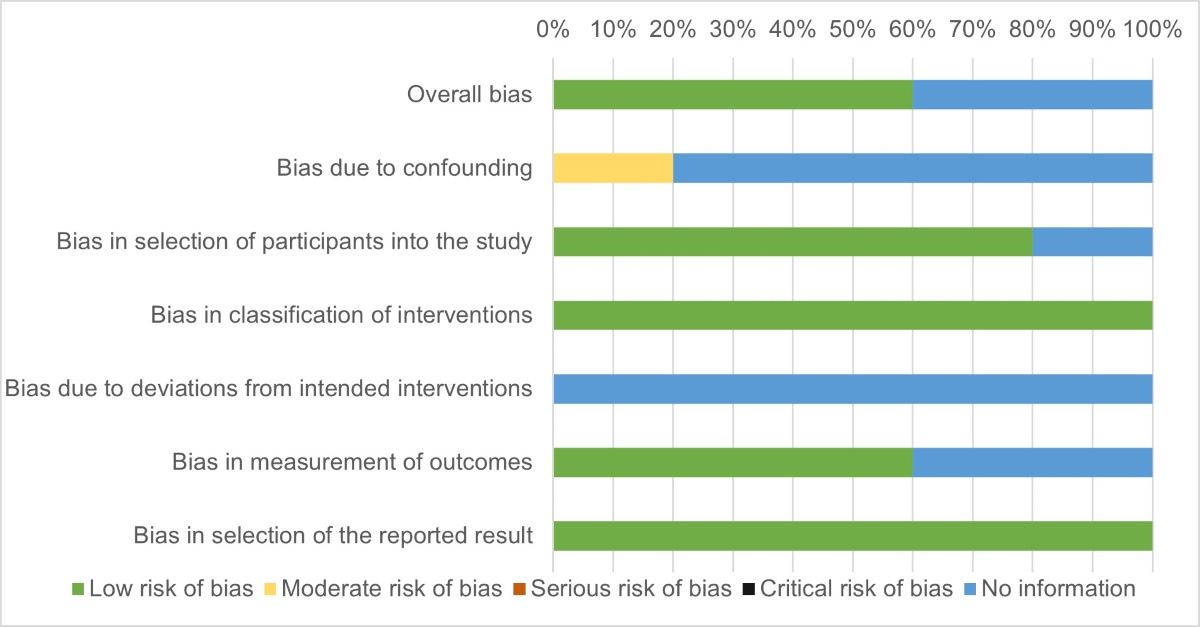

Supplement: Multimedia Appendix 8 [file jmir_v26i1e55757_app8.png]
