# Supplementary material for: Digital Interventions for Self-Management of Type 2 Diabetes Mellitus: Systematic Literature Review and Meta-Analysis
Source: J Med Internet Res. 2024 Jul 22;26:e55757. doi: 10.2196/55757 (PMC11301119; doi:10.2196/55757)
Supplement: Multimedia Appendix 6 [file jmir_v26i1e55757_app6.docx]

**Multimedia Appendix 6**

| **Author** | **Year** | **Group** | **Glucose data acquisition** | **Glucose data visualization** | **Telemedicine** | **Education** | **Intervention category** | **Comment** |
| --- | --- | --- | --- | --- | --- | --- | --- | --- |
| Allen et al. [[36](#_ENREF_36)] | 2011 | Intervention | CGM | Color graphs, summary tables of glucose excursions | One-time CGM counseling | Problem-solving skills | 1. High intensity |  |
|  |  | Control | CGM | Color graphs, summary tables of glucose excursions | One-time CGM counseling | General diabetes education |  |  |
| Amante et al. [[41](#_ENREF_41)] | 2021 | Intervention | SMBG | NA* | Care team contact triggered by SMBG reading | 7 self-care behaviors | 1. High intensity | Crossover trial using Livongo |
|  |  | Control | SMBG | NA | Usual care (no telemedicine) | NA |  |  |
| Athinarayanan et al. [[31](#_ENREF_31)] | 2019 | Intervention | SMBG | Web-based app | Web-based app to communicate with the care team | Nutrition education | 2. Medium intensity |  |
|  |  | Control | NA | NA | NA | NA |  |  |
| Azelton et al. [[20](#_ENREF_20)] | 2021 | Intervention | SMBG | Color-coded instant SMS | Healthy at Home coaches weekly phone calls and SMS, daily SMBG prompts | The team provided written and verbal explanations of the study | 1. High intensity |  |
|  |  | Control | NA | NA | NA | The team provided written and verbal explanations of the study |  |  |
| Cox et al. [[25](#_ENREF_25)] | 2021 | Intervention 1 | CGM | Device display | NA | 90 min group sessions focused on reducing post nutrient glucose excursions | 2. Medium intensity | All intervention groups were merged Together for analysis |
|  |  | Intervention 2 | SMBG | Device display | NA | 90 min group sessions focused on reducing post nutrient glucose excursions |  |  |
|  |  | Control | NA | NA | NA | Six 60 min weight loss sessions |  |  |
| Das et al. [[44](#_ENREF_44)] | 2018 | Intervention | SMBG | Device display | Counselling by health coach on medication, diet and exercise | In‑clinic physician and diet consultation | 2. Medium intensity |  |
|  |  | Control | NA | NA | NA | NA |  |  |
| Fantasia et al. [[47](#_ENREF_47)] | 2021 | Intervention | CGM | CGM-enhanced eConsult | NA | Patients asked to complete a diet and medication log while wearing the sensor | 3. Low intensity | FreeStyle Libre Pro |
|  |  | Control | NA | NA | NA | NA |  |  |
| Guo et al. [[26](#_ENREF_26)] | 2021 | Intervention | CGM | Real-time blood glucose values and a dynamic trend graph | Communicate with a GP and obtain personalized intervention programs | Health education once a week for 55 –65 minutes | 1. High intensity |  |
|  |  | Control | NA | NA | NA | Public health education material for T2DM were given out |  |  |
| Hee-Sung et al. [[40](#_ENREF_40)] | 2007 | Intervention | SMBG | Web-based glucose chart | SMS - nurse sent recommendations back to each patient | Before the intervention, each patient was instructed about the input of the website | 2. Medium intensity |  |
|  |  | Control | NA | NA | NA | In-clinic recommendations about medication, medication dosage and life style modification |  |  |
| Hsu et al. [[39](#_ENREF_39)] | 2016 | Intervention | SMBG | Diabetes care plan is visualized for the patient on the tablet computer application | Text messages with HCPs | The shared decision-making interfaces include weekly charts to help the subjects and HCPs | 2. Medium intensity |  |
|  |  | Control | NA | NA | NA | As telephone/fax communication with educators and physicians |  |  |
| Jeong et al. [[37](#_ENREF_37)] | 2018 | Intervention | SMBG | SMS based on glucose readings | Videoconferencing with endocrinologist |  | 2. Medium intensity |  |
|  |  | Control | SMBG | Device display | Outpatient appointments with their doctors | Education on standard diabetes self-care |  |  |
| Ji et al. [[28](#_ENREF_28)] | 2019 | Intervention | SMBG | Device display | Nurse case manager followed-up participants at least once a month, for 30‐minute sessions | Diabetes self‐management education + educational video to introduce diabetes self-management information including diabetes knowledge, diet/exercise education, insulin injection, and self‐monitoring of blood glucose levels. | 3. Low intensity |  |
|  |  | Control | NA | NA | NA | Diabetes self‐management education |  |  |
| Kim et al. [[23](#_ENREF_23)] | 2003 | Intervention | SMBG | Device display | Telephone intervention was provided to the intervention group by one of the researchers, a nursing PhD student, for 12 weeks. The frequency of telephone calls averaged 16 times for each individual, and the duration of each session was an average of 25 minutes. | The diabetes care booklet and a daily log, developed by the researchers for the study, were introduced. | 2. Medium intensity |  |
|  |  | Control | NA | NA | NA | Routine care (visiting a physician every 3 months) |  |  |
| Kirk et al. [[34](#_ENREF_34)] | 2009 | Intervention | In clinic | NA | Follow-up phone calls at 1 and 3 months | Written physical activity pack + in person delivered physical activity consultation | 3. Low intensity | Physical activity levels were measured using accelerometer |
|  |  | Control | In clinic | NA | Follow-up phone calls at 1 and 3 months | Written physical activity pack |  |  |
| Layne et al. [[35](#_ENREF_35)] | 2021 | Intervention | CGM | Mobile app | Remote lifestyle coaching and video consultations with endocrinologists | Onduo training | 1. High intensity | Onduo Virtual Diabetes Clinic |
|  |  | Control | NA | NA | NA | NA |  |  |
| Lee et al. [[30](#_ENREF_30)] | 2018 | Intervention | SMBG | Mobile app | The healthcare professionals, consisting of an endocrinologist, a nurse, and a dietitian, analyzed the transmitted records and sent messages on the secured website twice a week. | Usual dietary, nutritional, and exercise recommendations | 2. Medium intensity |  |
|  |  | Control | NA | NA | SMS regarding seasonal and health information without individualized message feedback by healthcare professionals | Usual dietary, nutritional, and exercise recommendations |  |  |
| Lee et al. [[38](#_ENREF_38)] | 2020 | Intervention | SMBG | Device display | TG participants received automated feedback on their glycemic and metabolic results. | Group-based diabetes education, dietary counselling, and medication reviews by the pharmacists | 2. Medium intensity |  |
|  |  | Control | SMBG | Device display | NA | Group-based diabetes education, dietary counselling, and medication reviews by the pharmacists |  |  |
| Lee et al. [[43](#_ENREF_43)] | 2019 | Intervention | CGM | CGMS result graph | NA | Individual education on diabetes + Guidelines on Diabetes Management booklet + CGMS result counseling and individualized Pattern Management | 2. Medium intensity |  |
|  |  | Control | NA | NA | NA | Individual education on diabetes + Guidelines on Diabetes Management booklet |  |  |
| McKenzie et al. [[46](#_ENREF_46)] | 2017 | Intervention | SMBG | Subjects were instructed to monitor and report glucose level via the Web | Personal health coach available daily via one-on-one texting for advice and problem solving | Weekly 90-minute group-based classes or Web-based recorded educational content | 1. High intensity | Sponsored by Virta Health, non-randomized parallel arm study. Two arms: all participants vs. the completers |
|  |  | Control | SMBG | Subjects were instructed to monitor and report glucose level via the Web | Personal health coach available daily via one-on-one texting for advice and problem solving | Weekly 90-minute group-based classes or Web-based recorded educational content |  |  |
| Nagrebetsky et al. [[42](#_ENREF_42)] | 2013 | Intervention | SMBG | Real-time graphical feedback on a mobile telephone | Remote nurse monitoring using a Web-based tool | Lifestyle-focused intervention consisting of monthly telephone calls, during which diet, physical activity, and recent changes in medication were discussed | 2. Medium intensity |  |
|  |  | Control | NA | NA |  | Lifestyle-focused intervention consisting of monthly telephone calls, during which diet, physical activity, and recent changes in medication were discussed |  |  |
| Odnoletkova et al. [[24](#_ENREF_24)] | 2016 | Intervention | SMBG | Device display | Five telephone sessions of a mean (range) duration of 30 (10–45) min, delivered at a mean (range) interval of 5 (3–8) weeks by a certified diabetes nurse educator | Participants were instructed on how to perform self-monitoring of blood glucose and interpret the results and were advised on the measurement frequency. | 3. Low intensity |  |
|  |  | Control | NA | NA | NA | Diabetes education by a certified diabetes educator and an annual consultation with an endocrinologist |  |  |
| Parsons et al. [[27](#_ENREF_27)] | 2019 | Intervention 1 | SMBG | Accu-Chek 360° View Tool | Contacted by telephone at an agreed time each month by their study nurse to review the previous months’ BG readings and related events | General diabetes education was provided by a study nurse + structured SMBG training | 2. Medium intensity |  |
|  |  | Intervention 2 | SMBG | Accu-Chek 360° View Tool | NA | General diabetes education was provided by a study nurse + structured SMBG training |  |  |
|  |  | Control | NA | NA | NA | General diabetes education was provided by a study nurse |  |  |
| Pimazoni-Netto et al. [[29](#_ENREF_29)] | 2011 | Intervention | SMBG | Accu-Chek 360° View Tool | NA | 10h of education and training by physicians, nurses, nutritionists, psychologists, and physical therapist over weeks 0-6 | 2. Medium intensity |  |
|  |  | Control | SMBG | Accu-Chek 360° View Tool | NA | 2h of general diabetes education at week 0 |  |  |
| Quinn et al. [[45](#_ENREF_45)] | 2011 | Intervention 1 | SMBG | Mobile app | Automated real-time messaging + patient web portal with direct contact to HCP + decision support | ADA guidelines | 1. High intensity |  |
|  |  | Intervention 2 | SMBG | Mobile app | Automated real-time messaging + patient web portal with direct contact to HCP | ADA guidelines |  |  |
|  |  | Intervention 3 | SMBG | Mobile app | Automated real-time messaging | ADA guidelines |  |  |
|  |  | Control | NA | NA | NA | ADA guidelines |  |  |
| Turnin et al. [[21](#_ENREF_21)] | 2021 | Intervention | SMBG | Device display | Monitoring interface linking TMG participants with investigators was designed as a secure web platform | Three tele-educational software programs were given to participants in the TMG: Nutri-Kiosk; Acti-Kiosk; Nutri-Educ | 2. Medium intensity |  |
|  |  | Control | NA | NA | NA | Face-to-face consultations in accordance with standard practices |  |  |
| Wayne et al. [[22](#_ENREF_22)] | 2015 | Intervention | SMBG | Connected Wellness Platform provided by NexJ Systems | Participants could communicate with their health coach at any time in the 24-hour cycle via secure messaging, or scheduled phone contact | Study explanation | 1. High intensity |  |
|  |  | Control | SMBG | Device display | Health coach phone contacts | Study explanation |  |  |
| Welch et al. [[32](#_ENREF_32)] | 2015 | Intervention | SMBG | Appraise Home HbA1c Kit; Heritage Labs International LLC | Internet based “diabetes dashboard” disease management tool | Five, in person, one-on-one diabetes education visits with a diabetes nurse or diabetes dietitian, | 2. Medium intensity |  |
|  |  | Control | SMBG | Appraise Home HbA1c Kit; Heritage Labs International LLC | NA | Series of individual patient visits with education content |  |  |
| Yang et al. [[33](#_ENREF_33)] | 2020 | Intervention | SMBG | Device display | Short feedback messages via a password-protected staff website at least once a week | All study participants of the intervention and control groups visited the outpatient clinic and received face-to-face consultations for individual management target of risk factors | 2. Medium intensity |  |
|  |  | Control | NA | NA | NA | All study participants of the intervention and control groups visited the outpatient clinic and received face-to-face consultations for individual management target of risk factors |  |  |
